# Characteristics of the Coronavirus Disease 2019 and related Therapeutic Options

**DOI:** 10.1016/j.omtm.2020.06.013

**Published:** 2020-06-24

**Authors:** Boxuan Huang, Rongsong Ling, Yifan Cheng, Jieqi Wen, Yarong Dai, Wenjie Huang, Siyan Zhang, Xifeng Lu, Yifeng Luo, Yi-Zhou Jiang

**Affiliations:** 1 Institute for Advanced Study, Shenzhen University, Shenzhen 518052, Guangdong, China; 2 Department of Physiology, Shenzhen University Health Science Center, Shenzhen Key Laboratory of Metabolism and Cardiovascular Homeostasis, Shenzhen University, Shenzhen 518071, China; 3 Department of Pulmonary and Critical Care Medicine, The First Affiliated Hospital of Sun Yat-sen University, Guangzhou 510080, China

## Abstract

The coronavirus disease 2019 (COVID-19) is a new type of pneumonia caused by severe acute respiratory syndrome coronavirus-2 (SARS-CoV-2) infection. COVID-19 is affecting millions of patients, and the infected number keeps increasing. SARS-CoV-2 is highly infectious, has a long incubation period, and causes a relatively high death rate, resulting in severe health problems all over the world. Currently there is no effective proven drug for the treatment of COVID-19; therefore, development of effective therapeutic drugs to suppress SARS-CoV-2 infection is urgently needed. In this review, we first summarize the structure and genome features of SARS-CoV-2 and introduce its infection and replication process. Then, we review the clinical symptoms, diagnosis, and treatment options of COVID-19 patients. We further discuss the potential molecular targets and drug development strategies for treatment of the emerging COVID-19. Finally, we summarize clinical trials of some potential therapeutic drugs and the results of vaccine development. This review provides some insights for the treatment of COVID-19.

## Graphical Abstract

**Figure:**
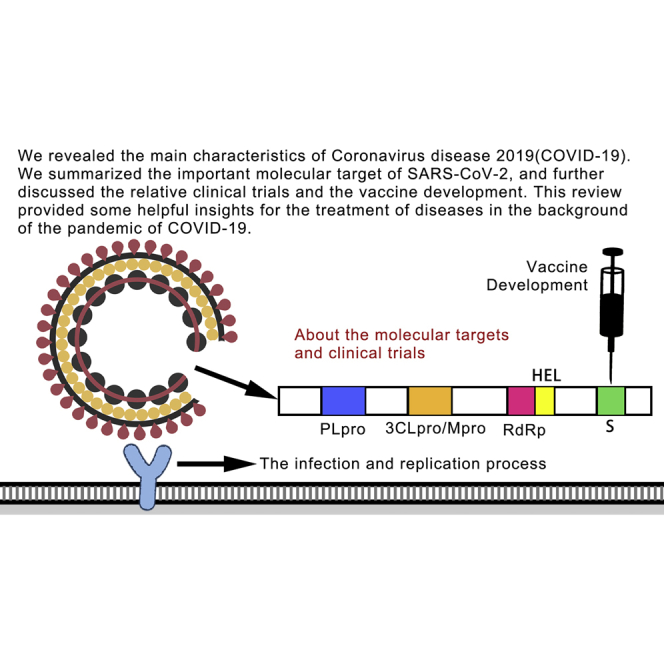

## Teaser

With the global pandemic of COVID-19, the development of effective therapeutic drugs and options has become more urgent. In this review, Boxuan Huang et al. mainly focus on the characteristics of COVID-19, the key molecular targets of SARS-CoV-2, relative drug clinical trial results, and progress in vaccine development.

## Main Text

The coronavirus disease 2019 (COVID-19) caused by severe acute respiratory syndrome coronavirus-2 (SARS-CoV-2) is now affecting millions of patients all over the world as of May 30, 2020.^1^^,^^2^ According to World Health Organization (WHO) statistics on March 3, the mortality rate among confirmed COVID-19 cases was 3.4%. As of May 22, according to Worldometer, the mortality rate is nearly 5.9%. In Italy, however, the mortality rate is more than 13%. The SARS-CoV-2 coronavirus is a type of single-stranded RNA virus that belongs to the coronaviruses family.^2^, ^3^, ^4^ Coronaviruses can be divided into four genera: *Alphacoronavirus* (αCoV), *Betacoronavirus* (βCoV), *Gammacoronavirus* (γCoV), and *Deltacoronavirus* (δCoV).^5^ Currently, seven coronaviruses are known to infect human, including two alphacoronaviruses (HCoV-229E and HKU-NL63) and five betacoronaviruses (HCoV-OC43, HCoV-HKU1, SARS-CoV, MERS-CoV, and SARS-CoV-2). During the past two decades, three previously unknown betacoronaviruses (SARS-CoV, MERS-CoV, and SARS-CoV-2) have emerged.^6^ These deadly coronaviruses cause lower respiratory tract infections, resulting in acute pneumonia, respiratory distress, cytokine storms, multiple organ dysfunctions, and even patient death.^1^^,^^7^^,^^8^

In this review, we highlight the pandemic of the emerging COVID-19, review the key molecular and clinical characteristics of SARS-CoV-2, and discuss the potential options for developing drugs for the treatment of COVID-19.

### Genomic Structure and Viral Protein Characteristics of SARS-CoV-2

**Figure 1:**
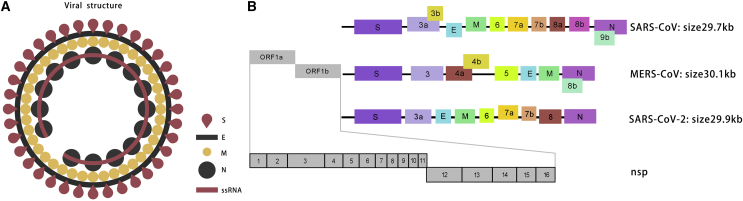
Genomic and Structural Characteristics of SARS-CoV-2 (A) The structure of SARS-CoV-2. S, proteins spike; E, envelope; M, membrane; N, nucleocapsid; ssRNA, single-stranded RNA. (B) The genomic characteristics SARS-CoV-2, SARS-COV, and MERS-COV. nsp, nonstructural protein.

The genome of SARS-CoV-2 contains 29,903 nt (NCBI: NC_045512.2), of which the GC content is 38%. The SARS-CoV-2 genome encodes about 9,860 aa. Similar to other coronaviruses, the SARS-CoV-2 genome consists of two flanked untranslated regions (UTRs), a 5′ long open reading frame (ORF1a/b) that encodes polyproteins, and several structural protein-encoding ORFs (Figure 1).^9^, ^10^, ^11^ The polyprotein encoded by 5′ ORF1a/b is cleaved by papain-like cysteine protease (PLpro) and 3C-like serine protease (3CLpro or main protease [M^pro^]). This process produces 16 nonstructural proteins (NSPs), including nsp3, nsp5, nsp12 (RNA-dependent RNA polymerase [RdRp]), nsp13 (helicase), and other NSPs that may be involved in viral transcription and replication.^9^^,^^10^ Additionally, the 3′ ORFs encode structural proteins spike (S), envelope I, membrane (M), and nucleocapsid (N). It has been reported that the ORFs of SARS-CoV-2 share high similarity with SARS-CoV.^9^^,^^10^ Also, the main differential regions between SARS-CoV-2 and SARS-CoV genomes are located in the ORF3b, S protein, and ORF8, of which thel S protein and ORF8 region were previously reported to be recombination hotspot regions.^10^^,^^12^, ^13^, ^14^

### The Transmission and Infection Process of SARS-CoV-2

Similar to SARS-CoV, SARS-CoV-2 also uses angiotensin converting enzyme II (ACE2) as a cellular entry receptor, suggesting that the infection process of SARS-CoV-2 into cells could be similar to that of SARS-CoV.^9^^,^^14^^,^^15^ Coronavirus enters into the host cells through the endosomal or lysosomal pathway in a proteolysis-dependent manner.^16^ The S protein of the coronavirus interacts with ACE2 protein on the host cells. Then, the S protein is cleaved into S1 and S2 subunits. The fusion peptide (FP) domain of S2 subunits is embedded in the host cell membrane, and the transmembrane (TM) domain of the S2 protein sub-type is embedded into the virus. After that, a hexapolymer hairpin structure is formed with the FP-HR1 domain and the TM-HR2 domains, which closes the spatial distance between the host cell and the virus and facilitates the membrane fusion and virus entry.^17^ A recent study compared the affinity between SARS-CoV-2 and SARS-CoV S proteins to the receptor ACE2 and revealed that the affinity between SARS-CoV-2 to ACE2 is 10- to 20-fold higher than that of SARS-CoV.^14^ This might explain the higher infectious capacity and widespread outcome of SARS-CoV-2.

The known transmission pathways of SARS-CoV-2 in humans include the following: (1) inhaling tiny droplets carrying virus, (2) close contact with virus carriers, (3) contact with a surface contaminated by SARS-CoV-2, and (4) aerosol transmission.^18^ Additionally, the latest research showed that in animals that are in close contact with humans, SARS-CoV-2 can efficiently replicate in cats, and the virus transmits in cats via respiratory droplets.^19^^,^^20^ Serological studies revealed that cats owned by COVID-19 patients had the highest neutralization titer for SARS-CoV-2. These studies pointed out the risk of cats involved in the transmission of SARS-CoV-2.^19^, ^20^, ^21^ Therefore, it is important that people and pets keep an appropriate distance.

### The Replication and Amplification Processes of SARS-CoV-2

Considering the genomic structure and other characteristics of SARS-CoV-2, its replication and amplification processes should be similar to other coronaviruses such as SARS-CoV.^11^^,^^22^^,^^23^ After the membrane fusion, the viral RNA genome is released into the cytoplasm of the host cells. Then, the ORF1a/b is translated into polyproteins 1a and 1ab (pp1a/pp1ab), which are cleaved into 16 NSPs.^11^^,^^22^^,^^23^ Many of the NSPs form the replicase-transcriptase complex (RTC) to replicate the genomic RNA. The full-length positive chain of genomic RNA is transcribed into a full-length negative chain template for synthesizing new genomic RNA and overlapping subgenomic negative chains, and then synthesizing and translating subgenomic mRNAs.

**Figure 2:**
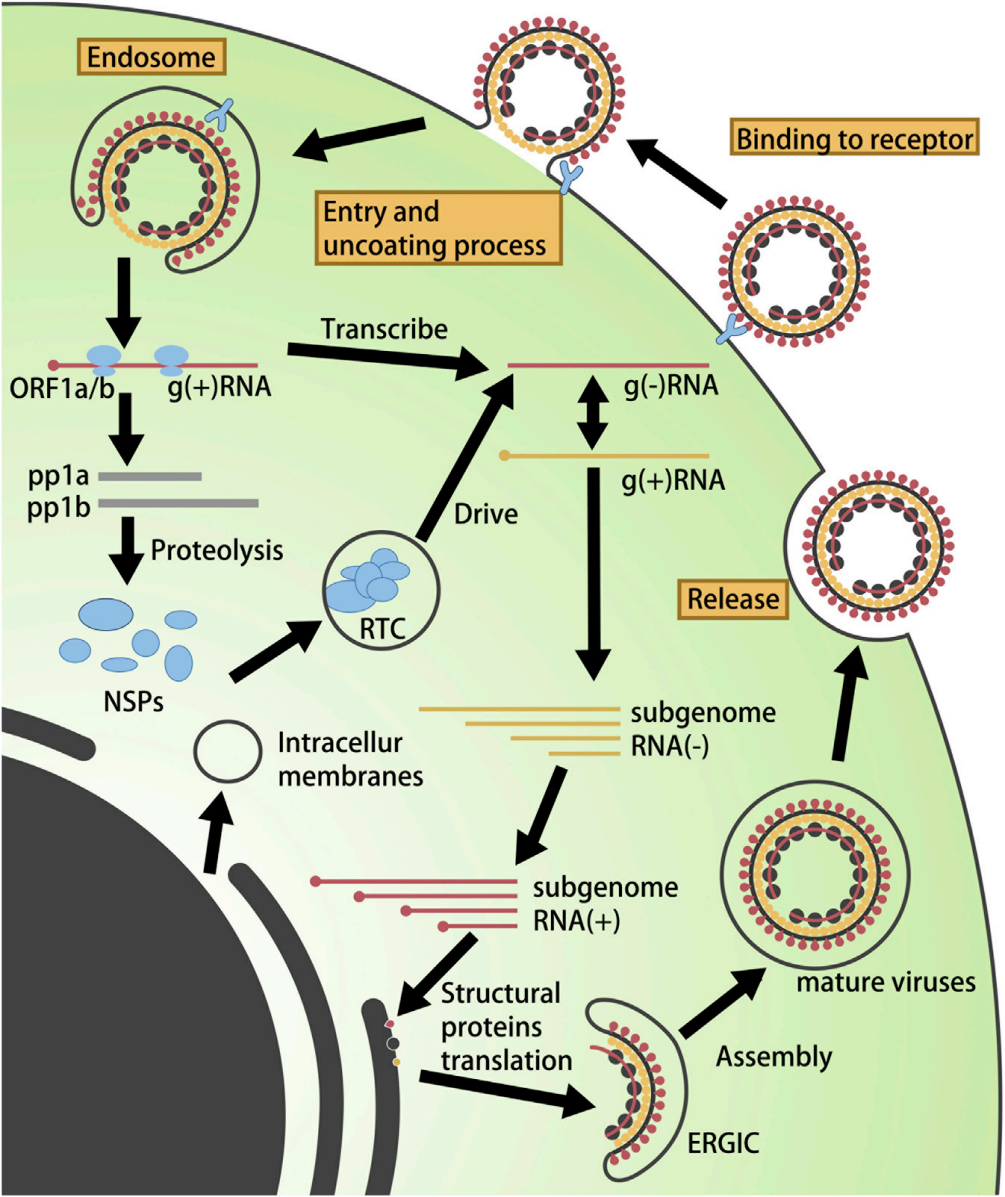
The Infection and Replication Process of SARS-CoV-2

After RNA replication, the structural proteins N, S, E, and M are translated. S, E, and M proteins insert into the endoplasmic reticulum (ER) and move to the endoplasmic reticulum-Golgi intermediate compartment (ERGIC) to form the mature viruses with the viral genome and N protein. After that, viruses are transported to the cell surface and then released out of the cells by exocytosis (Figure 2).^11^^,^^22^^,^^23^

### Diagnosis and Clinical Symptoms of SARS-CoV-2 Infection

The diagnosis of SARS-CoV-2 infection was based on nucleic acid detection.^24^, ^25^, ^26^ The mouth/nasopharyngeal swab samples or bronchoalveolar lavage fluid (BALF) samples were collected from the suspected patients and used for detection of SARS-CoV-2 with reverse transcriptase-polymerase chain reaction (RT-PCR). The nucleic acid detection is a multi-step method that involves RNA isolation, RT, and PCR with virus-specific primers. RNA could be degraded during clinical sample transfer and the RNA isolation process, leading to false-negative results. Also, in certain early-stage patients, the virus titer in the mouth/nasopharyngeal swab samples could be too low to be detected, which further increases the false-negative rates. According to a report, the positive rate of detection of COVID-19 using fluorescence quantitative RT-PCR as the detection method is only 30%–50%, which means it has a high false-negative rate. Also, studies have shown that thermal inactivation adversely affects the SARS-CoV-2 detection efficiency of RT-PCR, which is an important reason for the false-negative rate.^27^ In addition, immunoglobulin (Ig)G/IgM antibody detection is also important for the diagnosis of SARS-CoV-2 infection. In a report based on the antibody responses of 285 COVID-19 patients, approximately 17–19 days after the onset of symptoms, 100% of patients developed virus-specific IgG, while the proportion of patients with virus-specific IgM peaked at 94.1% after 20–22 days. Furthermore, titers of IgG/IgM antibodies tended to be stable within 6 days after seroconversion, which means that serological testing may be helpful for the diagnosis of suspected patients whose RT-PCR results are negative.^28^ Recently, a computed tomography (CT) scan was proposed to assist in the diagnosis of SARS-CoV-2 infection.^25^^,^^29^, ^30^, ^31^ CT scans revealed that SARS-CoV-2 infection causes bilateral pulmonary parenchymal ground-glass and consolidative pulmonary opacities in the lung. In addition, other features, including absence of lung cavitation, discrete pulmonary nodules, pleural effusions, and lymphadenopathy, could be discovered with CT scanning. Therefore, a CT scan provides a quick overview of the status and severity of the disease.^13^^,^^25^^,^^31^, ^32^, ^33^^,^^35^, ^36^, ^37^ However, CT images of SARS-CoV-2-infected lungs partially overlap with the images of other lung infectious diseases. Also, during the early stage of infection, patients might not have significant lung image changes. Therefore, the combination of nucleic acid detection and a CT scan is recommended for the precise detection of SARS-CoV-2 infection.^25^^,^^26^^,^^29^, ^30^, ^31^

The incubation period of SARS-CoV-2 ranges from 1 to 14 days (interquartile range, 2–7 days).^1^^,^^7^^,^^38^, ^39^, ^40^ Clinical symptoms of SARS-CoV-2 infection include fever, dry cough, and fatigue. More than 90% of the patients had fever, about 50%–76% patients had a cough, and around 25.3%–44% of the patients had fatigue symptoms.^1^^,^^2^ Other symptoms, which are not as common, include sputum production, rhinorrhea, sore throat, chest tightness, headache, vomiting, and diarrhea. Some patients only showed mild fatigue, low fever, no pneumonia, or even no symptoms. Clinically, based on the disease severity, patients can be divided into light, common, moderate, and critical condition groups.^1^^,^^7^^,^^13^^,^^41^ Most critical condition patients had breathing difficulties and/or hypoxemia. Additionally, the high incidence of multiple organ dysfunctions is one of the characteristics of COVID-19.^42^ In some severe cases, it can quickly progress into sepsis shock, acute respiratory distress syndrome, blood clotting dysfunction, and metabolic acidosis. In patients with coagulopathy, serological tests showed the existence of anticardiolipin IgA antibodies and anti-β_2_-glycoprotein IgA and IgG antibodies.^43^ However, the exact mechanisms that cause these symptoms remain to be explored, and the organ dysfunctions may be one of the causes of these symptoms. According to a study in New York City, most of the critically ill COVID-19 patients are associated with comorbidities, including hypertension, diabetes, chronic cardiovascular disease, and kidney disease. Additionally, the mortality rates of patients with these comorbidities are relatively high.^44^ The severity of SARS-CoV-2-infected patients is also associated with age, and the number of deaths is concentrated in people 40 years of age or older. Studies revealed that the morbidity of children and infants is less than in adults.^45^^,^^46^ This may be due to differences in the affinity between the receptor and the virus in different populations.^46^, ^47^, ^48^, ^49^

### Clinical Treatment of COVID-19

Currently there is no specific drug available to block SARS-CoV-2 infection or to kill the viruses. The treatment strategy is mainly determined by the clinical characteristics and severity of the disease, and different patients receive different treatments based on their conditions.^40^^,^^50^, ^51^, ^52^ Generally, patients are treated with strengthening support therapy to maintain sufficient caloric intake and water and electrolyte balance. Strategies including oxygen therapy, antiviral therapy, immunotherapy, organ support, and complication prevention are used for the prevention and control of acute respiratory distress syndrome, cytokine storms, organ failure, and secondary hospital infections. Also, based on the obvious abnormality of coagulation function in the clinical course of SARS-CoV-2 infection, Li et al.^50^ proposed early intravenous Ig and low-molecular-weight heparin anticoagulation therapy.^40^^,^^51^^,^^52^

Additionally, traditional Chinese medicines (TCMs) such as the Lianhua Qingwen capsule are widely used for the treatment of COVID-19 in China.^53^, ^54^, ^55^ TCMs can reduce fever symptoms, control the disease progression, decrease hormone use, and reduce complications in COVID-19 patients. Research confirmed that 13 natural compounds that exist in TCMs were found to have potential anti-SARS-CoV-2 activity.^56^ The application of TCMs helps to protect the functions of heart, liver, and kidney and enhance the patients’ immunologic function, therefore achieving therapeutic effects. Moreover, the combination of integrated Chinese and Western medicine has been proven to be an effective strategy for the prevention and treatment of COVID-19.^53^, ^54^, ^55^

### Potential Molecular Targets for the Therapy of COVID-19 and Strategies to Target SARS-CoV-2

**Figure 3:**
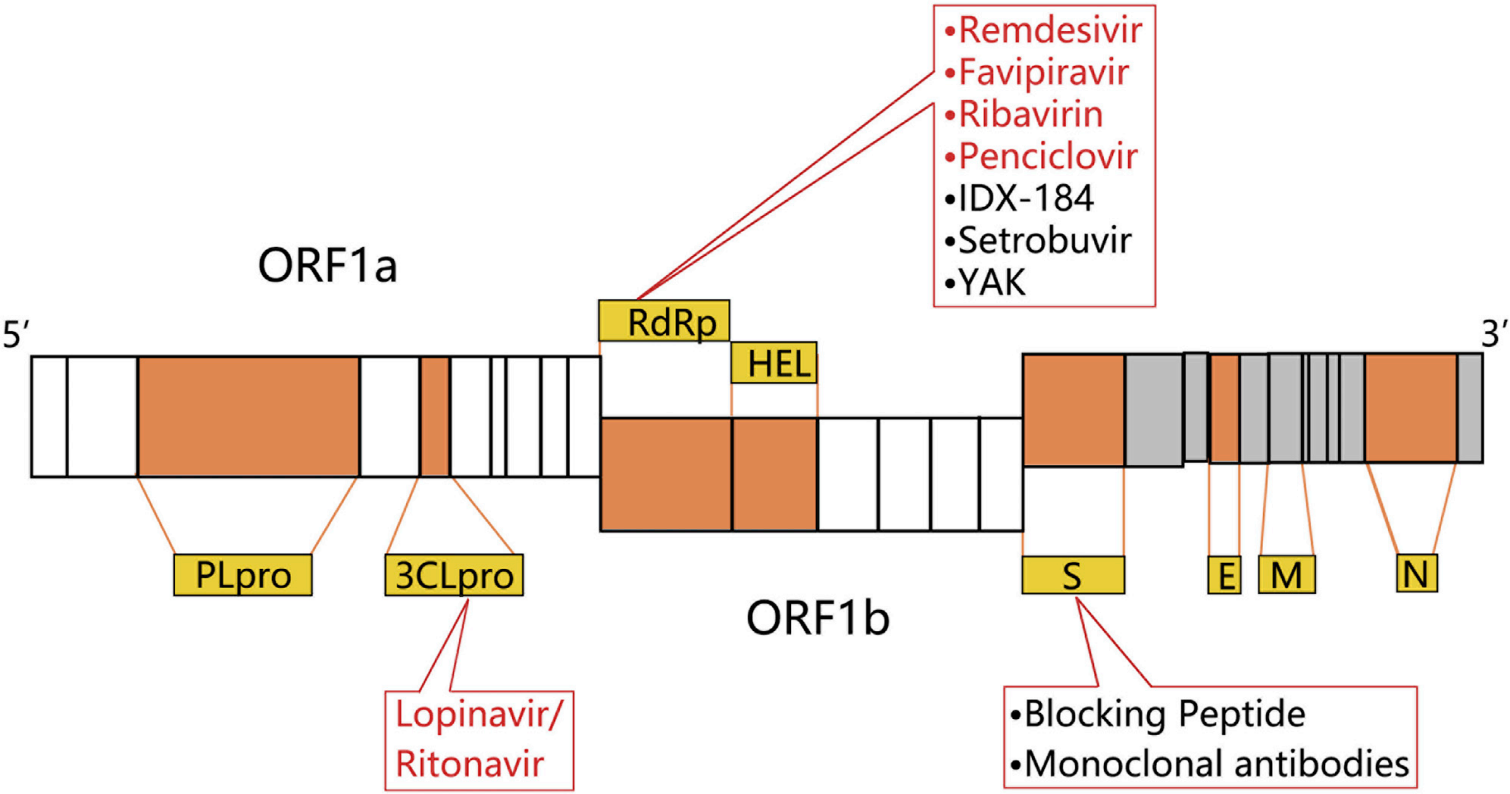
Potential Molecular Targets for the Therapy of COVID-19 The red text refers to drugs that are already under clinical trials. The black text in the box refers to potential drugs that are not yet under clinical trials. The gray boxes indicate the accessory genes.

Development of therapeutic drugs targeting SARS-CoV-2 infection or replication is an urgent need for the treatment of COVID-19.^57^, ^58^, ^59^, ^60^, ^61^ The potential therapeutic targets of COVID-19 include the following: (1) the NSPs: pp1a and pp1ab are cut by proteases (PLpro) and 3CLpro or M^pro^ to produce multiple NSPs, including RdRp, helicase, and nsp16. Inhibitors of these enzymes could block the replication of SARS-CoV-2.^4^^,^^11^^,^^17^^,^^62^ For example, the RdRp inhibitor remdesivir is currently under clinical trials for the therapy of COVID-19. Additionally, a molecular docking study on RdRp has revealed more potent drugs since they tightly bind to the RdRp of SARS-CoV-2. In addition, they found that guanosine derivative (IDX-184), setrobuvir, and YAK can be the top seeds for antiviral treatments.^63^ Moreover, through a large-scale computer-assisted drug screening, China’s joint scientific research team found that both saquinavir and ritonavir can inhibit the activity of SARS-CoV-2 M^pro^ and can also act as the nsp16 inhibitor.^64^ (2) The S protein: the S protein facilitates membrane fusion and virus entry by interacting with ACE2 protein on the host cells.^14^^,^^17^ It is reported that the affinity between SARS-CoV-2 to ACE2 is 10- to 20-fold higher than that of SARS-CoV, suggesting that blocking the S protein-mediated virus infection could be an effective strategy for COVID-19 treatment.^14^^,^^17^ Blocking peptides or monoclonal antibodies against S proteins are currently under investigation for their function in inhibiting SARS-CoV-2 infection.^65^ In addition, targeting the proteases, including PLpro or 3CLpro, could result in decreased expression of NSPs, therefore inhibiting the replication and infection of SARS-CoV-2 (Figure 3).^58^^,^^60^

Potential molecular targets of SARS-CoV-2 could provide strategies for drug screening and development. There are currently three main strategies to target SARS-CoV-2. Considering the urgent need for therapeutic drugs, the first and best strategy is to the test existing broad-spectrum antiviral drugs to assess the effects of these drugs on SARS-CoV-2.^6^^,^^57^^,^^66^ The advantage of testing the broad-spectrum antiviral drugs is that the safety of those drugs has been proven, and therefore if an antiviral drug can inhibit the replication or infection of SARS-CoV-2, it could be quickly applied for the clinical therapy of COVID-19.^67^ For example, the antimalarial drug chloroquine has broad-spectrum antiviral activity, although it cannot be regarded as a special effect, but it can be used as an effective drug.^67^, ^68^, ^69^, ^70^, ^71^, ^72^, ^73^, ^74^ At the same time, in TCM and natural products, there are some prescriptions that have a broad-spectrum inhibition effect for viruses, such as the Lianhua Qingwen capsule.^53^, ^54^, ^55^

The second method is to screen the existing bioactive compounds to identify small-molecule inhibitors or natural compounds for SARS-CoV-2.^6^^,^^57^^,^^66^ High-throughput screening of many easily available compounds is performed to screen for compounds that inhibit SARS-CoV-2 replication or infection. The main drawback of this approach is that while many of the identified drugs are active *in vitro* against coronaviruses, most are not suitable for clinical use. One reason is that they could be associated with immunosuppression, and another important reason is that their semi-maximum effective concentration (EC_50_) value may significantly exceed the peak serum concentration (C_max_) level at the therapeutic dose.^6^^,^^57^^,^^66^ Optimization of the lead compounds will be required to increase the efficacy and specificity of compounds for the therapy of COVID-19.

The third approach is to develop specific drugs based on the genome and protein characteristics of SARS-CoV-2.^6^^,^^57^^,^^66^ Based on the whole-genome sequencing of SARS-CoV-2, a series of sequence comparison and computational simulation results show that targeted drugs can be designed for the virus, including small interfering RNA (siRNA) molecules of specific viral enzymes involved in the virus replication cycle or antisense oligonucleotide (ASO), monoclonal antibodies against host receptors, and host cell protease inhibitors.^65^^,^^75^^,^^76^ Most of these drugs could have strong *in vitro* or *in vivo* anti-coronavirus activity, with limited side effects. Antisense oligonucleotides, monoclonal antibodies, and antiviral peptides are biologically targeted drugs, and their pharmacodynamics, pharmacodynamics, and side effects are easily characterized.^65^^,^^75^^,^^76^ In addition, these bio-targeted drugs have a short development cycle and can be used quickly in clinical settings.

In general, during the COVID-19 pandemic, the above methods can be used in combination to determine the best treatment options in time. Also, in the fight against the outbreak in China, the existing chemical/Chinese medicine and the bio-targeted drug are more used during outbreaks due to the short development cycle and the current urgent need for therapeutic drugs.

### Potential Therapeutic Drugs for COVID-19 Treatment

As discussed above, existing broad-spectrum antiviral drugs could be ideal therapeutic drugs for COVID-19 treatment. Herein, we review some of the potential therapeutic drugs that are under clinical trials to test their capacities to inhibit SARS-CoV-2 replication or infection. Remdesivir is a nucleotide analog with broad-spectrum antiviral activity, which is formally known as GS-5734. It is a RdRp inhibitor that was initially developed for treatment of Ebola virus-infected patients. A recent *in vitro* study revealed that remdesivir effectively inhibits SARS-CoV-2 in cells;^77^ therefore, at present, multiple clinical trials are ongoing to test its function for COVID-19 treatment. According to a recent report, which is based on data from severe COVID-19 patients treated with compassionate-use remdesivir from January 25 to March 7, 2020, clinical improvement was observed in 36 of 53 patients (68%), with one of its criteria being the oxygen-support class.^78^ Other RdRp inhibitors, including favipiravir, ribavirin, and penciclovir, could also be used as candidate therapeutic drugs due to their function in inhibiting the replication of coronaviruses.^77^ Hydroxychloroquine and chloroquine, immunosuppressive drugs previously approved for malaria treatment, have anti-inflammatory effects by impairing antigen presentation via the lysosomal pathway.^79^ Hydroxychloroquine and chloroquine indirectly reduce the production of anti-inflammatory cytokines, and it has been reported that chloroquine has shown apparent efficacy in treatment of COVID-19 *in vitro* and in patients.^67^^,^^69^^,^^71^^,^^77^^,^^80^, ^81^, ^82^

Lopinavir and ritonavir are protease inhibitors previously used to control HIV infection. It was shown that lopinavir/ritonavir administration significantly decreased SARS-CoV-2 viral loads in certain patients;^83^ however, recent clinical trials revealed no benefit of lopinavir/ritonavir treatment beyond standard care in other groups,^84^^,^^85^ and future trials might be required to confirm the therapeutic effect of lopinavir/ritonavir for COVID-19 patients. Arbidol (umifenovir) is a broad-spectrum antiviral approved for treatment of influenza and other respiratory viral infections. Arbidol induces interferon synthesis and inhibits the fusion between the viral capsid and the target cell membrane, which prevent viral entry into the target cell and therefore blocks virus infection.^86^, ^87^, ^88^ Treatment of COVID-19 patients with Arbidol combined with lopinavir/ritonavir results in an apparently more favorable clinical response than for the lopinavir/ritonavir-treated group, suggesting that Arbidol treatment could be beneficial for COVID-19 patients (Table 1).^89^ Moreover, there has been evidence that people with underlying diseases such as hypertension and other cardiovascular diseases have a higher critical rate after being infected with SARS-CoV-2.^90^ Renin-angiotensin system (RAS) dysfunction has been observed in COVID-19 patients. It was shown that patients using angiotensin-converting enzyme inhibitors (ACEIs) and angiotensin II type 1 receptor blockers (ARBs) had a lower rate of severe cases. The level of interleukin (IL)-6 in peripheral blood and peak viral load are decreased, and CD3 and CD8 T cell counts are increased, compared to other antihypertensive therapy. This evidence may be of use to reduce the mortality rate of patients with hypertension after infection with SARS-CoV-2.^91^

**Table 1:** List of Potential Therapeutic Drugs for the Treatment of COVID-19

Names	Target	Mechanisms	Current Statuses
Remdesivir	RdRp	nucleotide analog	shows efficacy in cells; under clinical trials
Favipiravir	RdRp	nucleotide analog	shows efficacy in cells; under clinical trials
Ribavirin	RdRp	nucleotide analog	shows efficacy in cells; under clinical trials
Penciclovir	RdRp	nucleotide analog	shows efficacy in cells; under clinical trials
Lopinavir/ritonavir	3CLpro	protease inhibitor	controversial results; under clinical trials
Hydroxychloroquine and chloroquine	endosomal acidification	disrupt intracellular trafficking and viral fusion events	show efficacy in cells and patients; under clinical trials
Arbidol	phospholipid	induces interferon synthesis and inhibits membrane fusion	shows efficacy in patients; under clinical trials

### Clinical Trials and Vaccine Development for COVID-19

Multiple clinical trials have been launched for potential therapeutic drugs that may be effective against COVID-19. As for remdesivir, the most recent report has indicated its clinical improvement for severe COVID-19.^78^ There are currently 16 clinical trials registered in ClinicalTrials.gov. The earliest two clinical trials related to this drug were launched in China. However, as the epidemic situation in China continues to improve, no eligible patients can be enrolled at present; accordingly, the trial of remdesivir in adults with mild and moderate COVID-19 (ClinicalTrials.gov: NCT04252664) has been suspended, and the trail for severe COVID-19 (ClinicalTrials.gov: NCT04257656) has been terminated. In a recent double-blind, randomized, placebo-controlled intravenous remdesivir trial of 1,063 adults hospitalized with COVID-19 and exhibiting symptoms of lower respiratory tract infection, remdesivir was superior to placebo in reducing recovery time.^92^ In another phase 3 trial of remdesivir in patients with severe COVID-19, Gilead announced that patients in both groups who received either a 10-day or a 5-day treatment course of remdesivir showed improved clinical status and no new safety signals were identified.

Although previous clinical trials showed no difference in clinical improvement time between the treatment with lopinavir/ritonavir and standard care,^84^^,^^85^ treatment with lopinavir/ritonavir is relatively safe and can significantly decrease SARS-CoV-2 viral loads in certain patients,^83^ so several clinical trials were conducted. In a randomized open-label phase 2 trial with a triple combination of interferon beta-1b, lopinavir/ritonavir, and ribavirin, when given within 7 days of symptom onset, this treatment was significant in reducing the shedding of SARS-CoV-2, compared with using lopinavir/ritonavir alone.^93^ As for chloroquine, a multinational registry analysis revealed that the use of hydroxychloroquine or chloroquine (with or without combination treatment with macrolide) was not beneficial to the treatment of patients infected with COVID-19; on the contrary, it increased the risk of ventricular arrhythmias and in-hospital death.^94^ Based on this, the WHO halted trials of hydroxychloroquine over safety fears. However, a multicenter prospective observational study showed that the proportion of patients receiving chloroquine for 10 and 14 days without detectable viral RNA was significantly higher than for the non-chloroquine group (91.4% and 95.9%, respectively, versus 57.4% and 79.6%, respectively). Additionally, most of these patients represented moderate cases, which revealed the therapeutic potential of chloroquine for early-stage patients.^95^ These findings indicate that before the widespread adoption of some drugs, the results of ongoing prospective, randomized, controlled studies are very important. In addition, the result of another prospective multicenter, open-label, randomized controlled trial on Lianhua Qingwen capsule revealed that it could be considered to ameliorate clinical symptoms of COVID-19.^96^ Although more than 300 clinical trials for COVID-19 are underway, there are no clinical data supporting any prophylactic therapy, and there are no randomized clinical trials data that any potential therapy can improve outcomes in COVID-19 patients yet.^13^^,^^97^

With the worldwide pandemic of COVID-19, the development for vaccines against COVID-19 becomes more urgent. On March 16, 2020, the first COVID-19 vaccine candidate entered human clinical trials. As of May 20, 2020, more than 120 candidate vaccines are under development (WHO data). For most of these candidates, the method is to block the S protein of SARS-CoV-2 by inducing neutralizing antibodies and prevent it from binding to the ACE2 receptor. There is an indication that vaccines could be available by early 2021.^98^ A phase 1 clinical trial of the first batch of vaccine (mRNA-1273) has been completed (ClinicalTrials.gov: NCT04283461). Additionally, Moderna announced positive interim phase 1 data for mRNA-1273 on May 18. The vaccine could induce the body to secrete neutralizing antibodies that effectively bind to antigens and block infection. All 45 subjects in three different dose groups achieved seroconversion 15 days after receiving the first shot and had detectable antibodies, and the researchers declared that mRNA-1273 was generally safe and well tolerated. Currently, the US Food and Drug Administration (FDA) has approved the mRNA-1273 vaccine to enter the fast track, and phase 2 clinical trials will begin soon. On May 22, *The Lancet* published the world’s first complete clinical phase 1 trial data (ClinicalTrials.gov: NCT04313127) for the COVID-19 vaccine. This is a vaccine that expresses the S protein of SARS-CoV-2 through a recombinant adenovirus type 5 (Ad5) vector. It was safe and tolerated in a total of 108 healthy adults in three groups, and could induce an immune response against SARS-CoV-2 in humans. The final results will be assessed within 6 months.^99^ Also, the vaccine is currently undergoing a phase 2 clinical trial. According to a report from Sinopharm, the inactivated anti-SARS-CoV-2 vaccine that they developed has been approved for phase 1 and phase 2 clinical trials by the National Medical Products Administration (NMPA) of China.

In the process of vaccine development, there are some difficulties that should be considered, such as the lack of animal models for *in vivo* drug efficacy evaluation, the higher mutation rate of coronavirus, as well as the possible antibody-dependent enhancement (ADE) effect in SARS-CoV-2.

### Perspective

The pandemic of COVID-19 has caused severe health problems all over the world. To slow down the increase of SARS-CoV-2-infected patients, superspreading events are non-negligible. According to a news report in *Science*, perhaps 10% of infected people caused 80% of the spread.^100^ Furthermore, it is important to avoid superspreading events by restricting gatherings of people. In addition, strategies including quarantine and personal protective equipment are essential to stop further spread of COVID-19. The rapid development of therapeutic drugs targeting SARS-CoV-2 is urgently needed for the treatment of current COVID-19 patients. For example, based on the highly conserved substrate-binding pocket among coronavirus M^pro^ (or 3CLpro), the combination of structure-based drug design, virtual screening, and high-throughput screening could help us find more effective anti-SARS-CoV-2 drug leads or treatment strategies.^101^ In the long-term, it is more important to develop vaccines against COVID-19 and provide active acquired immunity to COVID-19.

## Author Contributions

Conceptualization, Y.-Z.J.; Visualization, B.H. and Y.C.; Investigation, B.H., R.L., J.W., Y.D., and S.Z.; Writing – Original Draft, B.H., R.L., Y.C., J.W., Y.D., and S.Z.; Writing – Review & Editing, Y.-Z.J., X.L., and Y.L.; Funding Acquisition, Y.-Z.J.

## Conflicts of Interest

The authors declare no competing interests.
